# Impact of Rehabilitation Therapy on Disease Activity, Function, and Quality of Life in Rheumatoid Arthritis: A Systematic Review

**DOI:** 10.7759/cureus.92627

**Published:** 2025-09-18

**Authors:** Nimota Alapa, Barbara Tafuto, Rahul Mittal

**Affiliations:** 1 Health Informatics, Rutgers University, Piscataway, USA

**Keywords:** disease activity, dmards, physical function, quality of life, rehabilitation, rheumatoid arthritis

## Abstract

Rheumatoid arthritis (RA) is a chronic autoimmune disorder that leads to persistent joint inflammation and progressive disability. While treatment with disease-modifying anti-rheumatic drugs (DMARDs) remains the primary approach, rehabilitation therapies such as exercise and physical therapy provide long-term outcomes.

This systematic review evaluates the impact of rehabilitation interventions on RA patients receiving DMARD therapy compared with standard care without therapy.

Systematic literature research was conducted between January 2013 and July 2025 on PubMed, Cochrane Library, and Embase with predefined PRISMA guidelines. Randomized controlled trials, systematic reviews, and respective cohorts were included if they examined rehabilitation interventions, such as exercise or physical therapy, in addition to DMARDs, with outcomes related to disease activity, physical function, or quality of life. Risk of bias was assessed using Cochrane RoB 1.0 tools.

Eight randomized controlled trials involving 930 participants met the inclusion criteria. Most participants were women in their mid-50s to early 60s, with disease duration ranging from four to 16 years. Across studies, rehabilitation interventions were associated with meaningful improvements in physical function, reflected by reductions of 0.3 to 0.6 points in Health Assessment Questionnaire scores. Several studies also reported decreases of 0.4 to 0.7 points in Disease Activity Score (DAS28-ESR [erythrocyte sedimentation rate]/CRP [C-reactive protein]), particularly with multidisciplinary programs and structured exercise regimens. Quality-of-life measures similarly demonstrated improvement.

Rehabilitation to standard pharmacologic therapy may provide clinically significant benefits in disease control, functional capacity, and overall well-being. However, limitations included variability in reported outcomes and modest sample sizes, which restrict the generalizability of results. Overall, the evidence supports the incorporation of rehabilitation, particularly exercise and physical therapy, into standard care for RA patients as an effective complement to pharmacological management.

## Introduction and background

Rheumatoid arthritis (RA) is a chronic autoimmune condition that attacks the joints and causes them to become inflamed, painful, and stiff, impairing physical function and quality of life [1,2]. RA has the potential to cause extreme long-term disability and severely affect the patient's quality of life [2]. RA affects physical function and has systemic effects on other organs, such as the cardiovascular and respiratory systems [2,3]. RA attacks individuals of any age, but more so in females [4]. Its progressive nature leads to high societal burden, including reduced productivity, emotional distress, and long-term disability [1-7].

Interventions for RA typically include disease-modifying anti-rheumatic drugs (DMARDs) treatment [3,7]. The European Alliance of Associations for Rheumatology (EULAR) and the American College of Rheumatology (ACR) suggest the administration of DMARDs for disease activity management and prevention of joint damage, which are categorized into conventional synthetic DMARDs (csDMARDs) and biologic DMARDs (bDMARDs) [3,7]. According to EULAR and ACR, rehabilitation therapy, such as physical therapy and structured exercise, is also a significant addition to pharmacologic treatment and is beneficial for improving physical function, joint pain, and overall quality of life [8,9].

Rehabilitation interventions improve joint mobility and muscle strength, reduce systemic inflammation, support joint health and tendons, and potentially reduce inflammation [10-14]. Common strategies include strength and range-of-motion exercises, mind-body or aerobic activities, and multidisciplinary programs combining psychosocial care and physical and occupational therapy [11,12]. Rehabilitation interventions are safe overall and pose little risk; however, patients who have significant joint damage or significant comorbidities might experience a risk for overuse injury or symptom exacerbation if closely monitored [10].

Despite guidelines recommendations, rehabilitation remains underutilized, and supportive evidence of its effectiveness alongside DMARD therapy varies [12-15]. It is essential to consider the growing evidence that multidisciplinary care, both pharmacological and non-pharmacological treatment, is also essential to understand the impact of rehabilitation on disease activity, physical function, and quality of life for guiding integrated care options that optimize RA patients' long-term outcomes.

The patient population of interest in this review is adults with RA and, most importantly, patients who are already receiving DMARD treatment. This systematic review aims to evaluate the effectiveness of rehabilitation therapy and DMARD treatment in disease activity, physical function, and quality of life among RA patients. This review addresses the following research question: In patients with RA on DMARDs, how does the addition of rehabilitation interventions, compared to no rehabilitation, impact disease activity, physical function, and quality of life?

## Review

Methods

Where applicable, the Preferred Reporting Items for Systematic Reviews and Meta-Analyses (PRISMA) guidelines were used [16] for transparency and replicability. This review was registered in PROSPERO (Registration ID: CRD420251052312).

Eligibility criteria

The inclusion criteria for studies were as follows: (1) RA patients; (2) treatment with DMARDs, including csDMARDs or bDMARDs; (3) studies comparing rehabilitation, such as physical therapy or exercise groups, to usual care or a no-therapy control group; (4) use of DMARD at baseline and with changes after intervention; (5) adults and older adults with RA (age 18+ years); (6) focus on studies that measured disease activity scores, physical function, or quality of life; (7) only clinical trials (randomized or non-randomized, controlled, or non-controlled), and systematic reviews/meta-analyses for pearl growing [17] in the last 12 years to account for current advances in RA treatment; (8) only full-length papers in English.

Exclusion criteria were as follows: (1) patients diagnosed with a different type of arthritis; (2) studies on animals; (3) case reports, expert opinions, letters to the editor, and conference abstracts or publications; (4) studies that did not report relevant outcomes, i.e., studies that focused on pharmacokinetics only; (5) studies that did not report results on any of the outcomes (disease activity, physical function, and quality of life).

Search strategy

An initial literature search was done on January 18, 2025, with a final look-up search on July 21, 2025, through PubMed, Cochrane Library, and Embase using Boolean Equation (AND, OR), MeSH (Medical Subject Headings) terms, and free-text keywords of RA, bDMARDs, csDMARDs, and physical therapy, rehabilitation therapy, and exercise. Filters applied were publications from January 2013 up to July 2025, adults of 18+ years, human species, English language, and study types (randomized controlled trials [RCTs], non-randomized trials, and systematic reviews/meta-analyses). The search strategy for each database is provided in the Appendices (Table S5).

Study selection

The selected articles from each database were stored in EndNote for duplicates removal, and the rest of the articles were exported to Covidence (Veritas Health Innovation Ltd., Melbourne, Australia) for title and abstract review, full-text review, data extraction, and quality assessment. Initial screening, data extraction, and full-text review were done by the principal investigator. Uncertainty regarding eligibility was resolved by consulting with a second subject matter expert when necessary. Reference lists of included studies were also examined for additional eligible articles (“pearl growing”). The completed manuscript, including study selection, data extraction, and narrative synthesis, was reviewed by a subject matter expert to ensure accuracy and rigor.

Data collection

The following data were extracted by the principal investigator from the final selected articles in Covidence (Veritas Health Innovation Ltd., Melbourne, Australia): the type of study, country, the year of the study, the number of participants, study design, baseline characteristics, intervention outcomes such as frequency, duration, the intervention, patient characteristics, type of DMARD medication, disease activity, comparator, results measurement, time point, and statistical significance for disease activity, physical function, and quality of life. The extracted data were exported from Covidence to Microsoft Excel [18] to organize for a narrative synthesis of results. To ensure accuracy and reliability, all extracted data were reviewed by a subject matter expert, and any uncertainties were clarified through discussion.

Outcome measures

The primary outcome was disease activity measured by DAS-28 (erythrocyte sedimentation rate [ESR] or C-reactive protein [CRP], as reported in the study) scores, while the secondary outcome was assessing Health Assessment Questionnaire (HAQ) or Michigan Hand Outcome Questionnaire (MHQ) when reported, and quality of life using self-reported questionnaires. The principal investigator prioritized outcome measures based on clinical relevance to hand function, patient-oriented outcomes, and use in included trials. Using narrative synthesis, the measures comprehensively understood these interventions' impact on RA patients alongside DMARD treatment.

Data analysis

The outcomes of DMARD treatments were compared with or without rehabilitation through narrative synthesis. Effect sizes and statistical significance were reported where available. No meta-analysis was performed due to heterogeneity of interventions, outcome measures, and study designs.

Risk of bias

The risk of bias was assessed independently using the Cochrane Risk of Bias Tool (Rob 1.0). The principal investigator appraised the following areas: random sequence generation, allocation concealment, blinding of participants and personnel, blinding of outcome assessors, incomplete outcome data, selective outcome reporting, and other bias. Each area was assigned “low risk,” “high risk,” or “unclear risk” according to criteria set out in the Cochrane Handbook. RoB 1.0. [19] was used because the included studies predominantly predate the adoption of RoB 2.0, and the extracted data align with the standard 1.0 domains. Assessment was conducted by the principal investigator, with verification by a subject matter expert. 

Results

**Figure 1:**
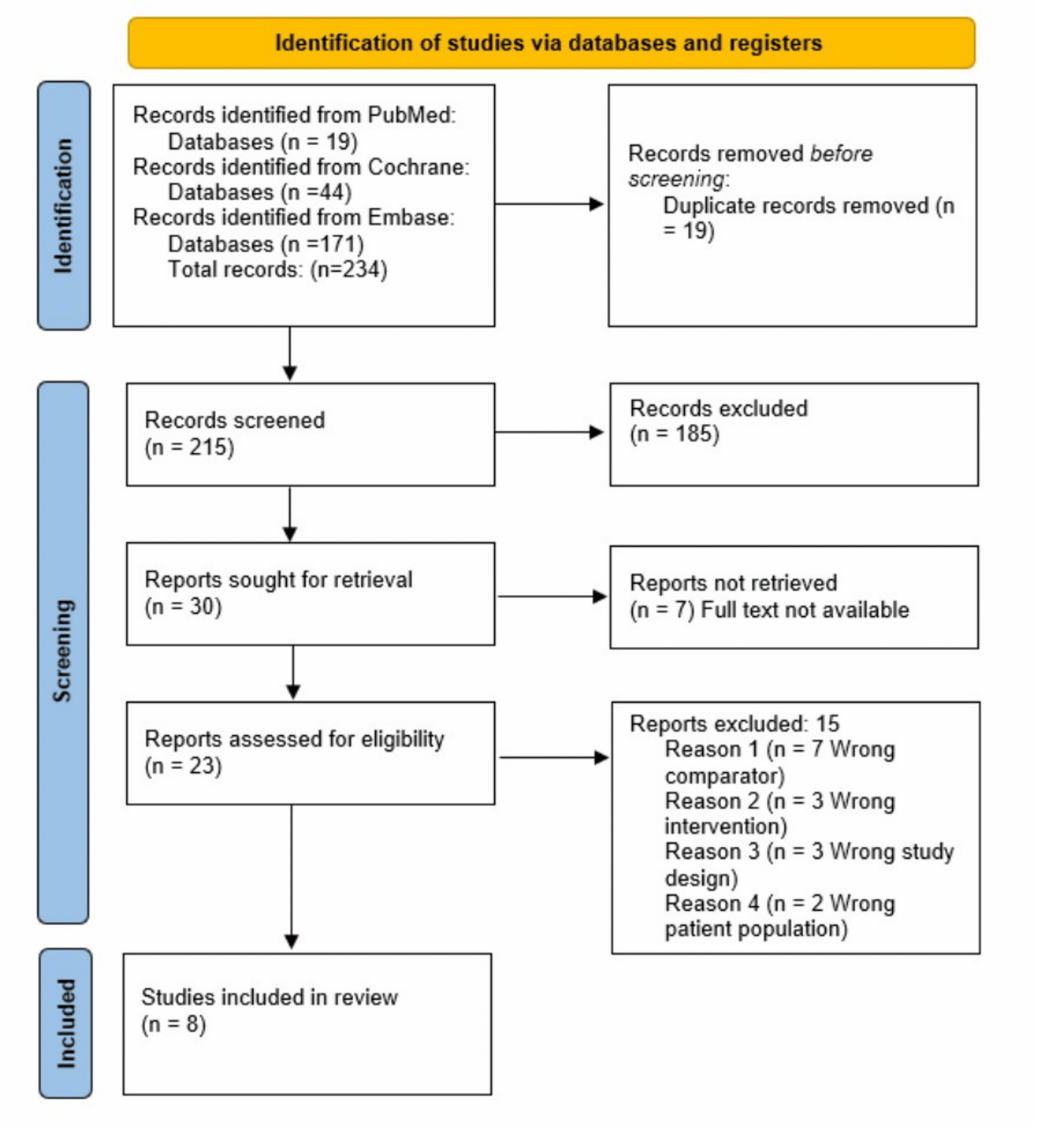
PRISMA Flow Diagram of the Study Selection Process

A total of 234 were screened for inclusion criteria after conducting a comprehensive search in PubMed, Cochrane Library, and Embase databases. Nineteen were identified from PubMed, 44 from Cochrane Library, and 171 from Embase, resulting in a final 215 articles identified after removing 19 duplicates; see Figure 1. Based on the assessment from the title and abstract screening, 185 articles were excluded, and the remaining 30 articles were selected for full-text article review. Seven were excluded due to the unavailability of the full-text articles. Twenty-three potential articles were reviewed; the final eight chosen articles met the inclusion criteria [20-27], and 15 were excluded, as shown in the PRISMA flow diagram (Figure 1).

Study characteristics of included articles

Table 1 summarizes the eight studies on RA interventions. A total of eight RCTs published between 2015 and 2024 evaluated the effects of incorporating various rehabilitation interventions alongside routine DMARD therapy in patients with RA. The intervention differed between studies, with the majority being exercise-based techniques [20,25], mind-body practices like Yi Jinjing [21] and yoga [26], galvanic electrotherapy [22], cervical stabilization exercises [23], aquatic aerobic exercise [27], and multidisciplinary care approaches [24]. Control groups did not receive any additional rehabilitation with DMARD treatment. Participants range from 33 to 490, with the majority being female.

The study summary in Table 1 provides additional information.

**Table 1:** Study Summary of Selected Articles DMARDs, disease-modifying anti-rheumatic drugs.

Title	Primary Author's Last Name	Year	Study Type	Total Participants	Intervention	Comparator
Benefits of exercise in patients with rheumatoid arthritis: a randomized controlled trial of a patient-specific exercise programme	Azeez [20]	2020	Randomized controlled trial	52	Routine DMARDs treatment + cardiovascular fitness and strength exercise	Routine DMARDs treatment and no exercise
Effect of traditional Chinese Yijinjing exercise on hand dysfunction in rheumatoid arthritis patients: a randomized controlled trial	Chang [21]	2024	Randomized controlled trial	66	Routine DMARDs treatment + Yijinjing exercise	Routine DMARDs treatment and no exercise
The effectiveness of galvanic electrotherapy and a conservative hand exercise program in a rheumatoid hand: A randomized controlled trial	Dülgeroğlu [22]	2016	Randomized controlled trial	33	Routine DMARDs treatment + galvanic electrotherapy	Routine DMARDs treatment and no exercise
							Effects of video-based cervical stabilization home exercises in patients with rheumatoid arthritis: a randomized controlled pilot study	Gulcemal [23]	2024	Randomized controlled trial	36	Routine DMARDs treatment + cervical stabilization exercise	Routine DMARDs treatment and no exercise
														Evaluation of a multidisciplinary care model to improve quality of life in rheumatoid arthritis: a randomised controlled trial	Lahiri [24]	2022	Randomized controlled trial	96	Routine DMARDs treatment + 6 multidisciplinary (rheumatologist, nurse, social worker, physiotherapist, occupational therapist, podiatrist) team	Routine DMARDs treatment and no exercise
																					Exercises to improve function of the rheumatoid hand (SARAH): a randomised controlled trial	Lamb [25]	2015	Randomized controlled trial	490	Routine DMARDs treatment + mobility and four strength or endurance exercises	Routine DMARDs treatment and no exercise
Effects of yoga in a daily life program in rheumatoid arthritis: A randomized controlled trial	Puksic [26]	2021	Randomized controlled study	57	Routine DMARDs treatment + yoga exercise	Routine DMARDs treatment and no exercise																					
							Effectiveness of aquatic exercises in women with rheumatoid arthritis a randomized, controlled, 16-week intervention—The HydRA Trial	Siqueira [27]	2017	Randomized, blinded, prospective, controlled clinical trial	100	Routine DMARDs treatment + water-based aerobic exercise (GW), land-based aerobic exercise (GL)	Routine DMARDs treatment and no exercise

Patient characteristics

The study's patient populations are also heterogeneous regarding age, sex, and disease duration. Some studies had fewer participants due to the inability to follow up. They were not included in the final analysis, and the studies also had most participants with median ages of the mid-50s to early 60s, with more female participants to mirror the gender predominance of RA as reported commonly. In Gulcemal et al. [23], for instance, the intervention group had a median age of 61 years, while the comparator had a median of slightly less at 55.5 years. In the same way as Lamb et al. [25], the intervention group had a mean of 61.3 years and a median disease duration of 10 years, while their comparator had a mean of 63.5 years and an exact disease duration. According to Azeez et al. [20] and Dülgeroğlu et al. [22], disease duration proved variable between studies, from a minimum of two years to more than 11 years. Still, most trials recruited established diseases that were seven years or longer. In all instances, intervention and comparator groups were well-balanced for age, gender distribution, and disease duration at baseline, enhancing the internal validity of the results. These studies had many participants who were females with disease durations of early to advanced stages of RA and with a broad representation of RA patients. Table 2 provides detailed information on the key patient characteristics of the eight studies.

**Table 2:** Summary of Patient Characteristics NR, not reported.

Primary Author's Last Name/Year	Intervention Group			Comparator Group		
	Age Median	Gender	Stage of Disease Years (Median) or (Mean ± SD)	Age Mean (SD)	Gender	Stage of Disease Years (Median) or (Mean ± SD)
Azeez [20]/2020	58.5	86% female	2	63	83% female	9
Chang [21]/2024	58.5	96.7% female	NR	63	93.3% female	NR
Dülgeroğlu [22]/2016	55	100% female	11	51.5	100% female	11
Gulcemal [23]/2024	61	71% female	(9.5 ± 6.99)	55.5	71% female	(7.0 ± 6.55)
Lahiri [24]/2022	56.6	91% female	5.7	56.5	81.2% female	5.3
Lamb [25]/2015	61.3	76% female	10 (4)	63.5	76% female	10 (4)
Puksic [26]/2021	52.9	100% female	7.4	57.9	89% female	8.7
Siqueira [27]/2017	54.5	100% female	Land (7.7 ± 2.9), water (9.2 ± 3.1)	53.2	100% female	(8.5 ± 4)

Rehabilitation training protocols

The assessed intervention showed significant heterogeneity concerning content, frequency, and duration in the following categories. Aerobic exercises: walking, cycling, swimming [20,27], typically 2-3 supervised sessions/week for 12-16 weeks, sometimes with additional home exercises. Mind-body practices: Yi Jinjing [21] and yoga [26], generally 12-week programs, with emphasis on flexibility, balance, and gentle strengthening. Specific rehabilitative techniques: galvanic electrotherapy combined with hand exercises [22] and cervical stabilization via video sessions [23], often short-term but high-frequency interventions. Multidisciplinary care: Integrated approaches involving multiple caregivers, targeting functional and psychosocial outcomes [24]. The heterogeneity mirrors the wide range of rehabilitative methodologies employed with RA, both low-intensity formats with high frequency. While detailed procedural descriptions are available for mind-body and electrotherapy interventions, other interventions, particularly multidisciplinary care programs, were described more superficially. This imbalance limits comparability across intervention types and precludes subgroup analysis to determine whether certain rehabilitation modalities are more effective than others.

Effect of rehabilitation intervention on RA

Eight studies assessed various outcomes and primarily used measures like Disease Activity Scale with 28-joint count (DAS-28), HAQ, Global Fatigue Index (GFI), Rheumatoid Arthritis Quality of Life (RAQoL), Short Form-12 (SF-12), and visual analog scale (VAS) to assess the effectiveness of the rehabilitation intervention on disease activity, quality of life, and functional status (see Table 3). Combined evidence across the included studies indicates that rehab intervention with usual DMARD therapy presents multifaceted patient benefits for RA, though variability occurs in the effect on inflammatory disease activity. For instance, Azeez et al. [20] reported no significant difference in DAS-28 scores after three months. In contrast, Chang et al. [21] and Siqueira et al. [27] reported significant DAS-28 score improvements after 12-week Yi Jinjing exercise (from 2.85 to 2.15, p=0.001) and 16-week aquatic-based aerobic workout (from 3.8 to 3.1, p < 0.05), illustrating direct disease activity influence. Multidisciplinary practice styles also registered modest, yet significant DAS-28 decreases [24]. Significantly, functional measures were better across most studies, with significant HAQ scale gains, MHQ among other measures of physical function [23,25]. Quality-of-life indicators also were favorable for intervention groups, with improvements for fatigue [20], RAQoL [23], as well as general health-related quality of life [24]. These findings emphasize the potential for rehabilitation to augment patient-oriented measures of function and well-being even when inflammatory markers or disease activity metrics exhibit small changes. Collectively, this evidence bolsters the incorporation of rehabilitation as an essential DMARD pharmacologic management in RA, with impacts on the physiology of the disease alongside the psychosocial elements of the disease.

**Table 3:** Study Outcomes Table DAS-28, Disease Activity Scale with 28-joint count; DAS28 CRP, Disease Activity Score 28 C-reactive protein; DAS28-ESR, disease activity in 28 joints using ESR; VAS, visual analog scale; MHQ, Michigan Hand Outcome Questionnaire; R, right; L, left; SF-12, Short Form-12; SF-36, Short Form-36; HAQ, Health Assessment Questionnaire; mHAQ, modified Health-Associated Questionnaire (8-item); HAQ-DI, Health Assessment Questionnaire Disability Index; RAQoL, Rheumatoid Arthritis Quality of Life Scale; RAPS, Rheumatoid Arthritis Pain Scale; GFI, Global Fatigue Index; EQ-5D-3L, European QOL-5-Dimension-3-Level; DHI, Duruoz Hand Index; RH, right hand; LH, left hand; NDI, Neck Disability Index; ESR, erythrocyte sedimentation rate; CRP, C-reactive protein; IQR, interquartile range; SD, standard deviation; NR, not reported.

First Author/Year	Intervention by Group	Frequency and Duration	Outcome Category	Outcome Measures	Results
Azeez et al., 2020 [20]	Intervention (I): Walking, cycling, swimming per patient preference. Strength training program for muscle and grip strength. Control (C): Standard care	I: 3 sessions over 3 months with the physiotherapist (initial + 2 follow-ups at 4-week intervals) plus home-based daily exercise	Disease Activity	DAS-28 (0-28) median (range) at baseline and 3 months. A low score indicates reduced RA activity.	There was no significant improvement in both groups at 3 months: DAS-28 (2.39 (0.49–3.70) to 2.19 (0.43–5.02)) (within-group p=0.409) compared to (2.38 (0.49–5.30) to 2.59 (0.63–5.91)) (within-group p=0.508).
			Quality of Life	GFI (1-50) median (range) at baseline and 3 months (high score indicates low performance)	There was a significant improvement in GFI from baseline (13.2 (6.4–34.1)) to 3 months (10.9 (6.5–37.5)) (within-group p=0.047) vs control baseline (24.8 (6.3–47.7)) to 3 months (24.8 (6.3–48.2)) (within-group p=0.96)
			Physical Function	HAQ (0-3) median (range), at baseline and 3 months (a low score indicates good performance)	There was a significant improvement in intervention HAQ from baseline to 3 months: 0.5 (0.0–2.4) to 0.25 (0.0–2.5) (within-group p=0.05) vs control 1.1 (0–3.0) to 0.80 (0.0–2.9) (within-group p=0.026).
Chang et al., 2024 [21]	I: Yi Jinjing exercise (a psychosomatic exercise based on Chinese medicine theory). C: Usual care	Three times a week for 12 weeks.	Disease Activity	DAS-28 ESR (0-28) means±SD at baseline and 3 months. A low score indicates reduced RA activity.	There was a significant improvement in both groups at 3 months: DAS-28 ESR (2.85±0.82 to 2.15±0.83) (within-group p=0.001) compared to control (2.97±0.85 to 2.83±0.67) (within-group p=0.621). Between-group p-value = 0.002
			Quality of Life	HAQ-DI (0-3) medians (25, 75%) at baseline, 3 months (high score indicates good performance)	There was a significant improvement in HAQ-DI from baseline (0.13 (0, 0.75)) to 3 months (0 (0, 0)) (within-group p=<0.001) vs control baseline (0.13 (0, 0.38)) to 3 months (0.13 (0, 0.5)) (within-group p=0.003) (between-group p=0.036).
			Physical Function	MHQ (0-100) means±SDs at baseline, 3 months (a low score indicates good performance)	There was no significant improvement in intervention MHQ from baseline to 3 months: 51.48±8.36 to 65.57±5.86 (within-group p-value <0.001) vs Control 49.58±10.48 to 55.04±7.57 (within-group p-value <0.001), p-value (between-group) <0.001.
Dülgeroğlu et al., 2016 [22]	I: Galvanic electrotherapy applied to both hands in a water tank for 20 min. C: Usual care	Three times per day for 10 days	Disease Activity	DAS-28 (0-28) median (Min-Max) at baseline and 8 weeks. A low score indicates reduced RA activity.	Only the baseline was reported. At baseline: 4.10 (2.10-5.60) vs control 3.53 (2.38-5.20); between-group p=0.244.
			Quality of Life	HAQ (0-3) median (Min-Max), at baseline and 8 weeks (a low score indicates good performance)	There was a significant improvement in HAQ from baseline (1.50 (0.12-2.30)) to 8 weeks (1.10 (0.00-2.30)) vs control from baseline (1.05 (0.15-2.50)) to 8 weeks (0.73 (0.00-2.10)).
			Physical Function	DHI (0-90) median (Min-Max), at baseline and 8 weeks (a low score indicates good performance)	There was a significant improvement in intervention DHI from baseline to 3 months: 17.50 (2.00-62.00) to 7.00 (0.00-49.00) vs control 17.50 (0.00-57.00) to 9.50 (0.00-34.00).
Gulcemal et al., 2024 [23]	I: Progressive cervical stabilization exercises targeting the deep neck flexor muscles were delivered to the patients as video messages. C: Usual care	3 times per week for 6 weeks	Disease Activity	DAS-28 CRP (0-10) median (IQR 25th/75th) at Week 1.	Low DAS-28 (≤3·2) reported at week 1 in both the intervention group and the control group: baseline 3.1 (2.1/4.0) vs control 3.2 (2.1/3.8). Between-group p=0.810
			Quality of Life	RAQoL (0-30) median (IQR 25th/75th) at Weeks 1 and 7 (a high score indicates low performance)	There was a significant improvement in intervention during Week 1: 19.0 (12.3/22.3) vs Week 7: 7.5 (3.0/12.0) within-group p=0.001 vs control during Week 1: 15.5 (3.3/25.0) vs Week 7: 15.5 (6.3/26.5); within-group p=0.170.
			Physical Function	HAQ (0-3) median (IQR 25th/75th), at Weeks 1 and 7 (high score indicates good performance)	There was a significant improvement in intervention HAQ during Week 1: 0.5 (0.2/1.3) vs Week 7: 0.1 (0.1/0.4) within-group p=0.001 vs control during Week 1: 0.6 (0.1/1.1) vs Week 7: 0.6 (0.2/1.4); within-group p=0.235.
Lahiri et al., 2022 [24]	Patient's visit to a 6-member multidisciplinary team of rheumatologist, physical therapy, occupational therapy, podiatrist, nurse, and medical social worker for 20 min per healthcare practitioner. C: Usual care	2 h spent on the clinic visit.	Disease Activity	DAS-28 ESR (0-28) mean (95% confidence interval) at baseline and 6 months. A low score indicates reduced RA activity.	There was a significant improvement in both groups at 3 months: DAS-28 ESR 3.13 (2.84, 3.42) to 2.79 (2.53, 3.06) within-group p=0.02 compared to control 2.80 (2.56, 3.04) to 2.90 (2.63, 3.17) within-group p=0.49 with between-group p=0.03.
			Quality of Life	EQ-SD-3L (-0.59 to 1) mean (95% confidence interval) at baseline, 6 months (high score indicates good performance)	There was a significant improvement in intervention from baseline 0.72 (0.65, 0.79) to 6 months 0.79 (0.72, 0.86) within-group p=0.06 vs control from baseline 0.76 (0.69, 0.83) to 0.73 (0.66, 0.81); within-group p=0.36 with between-group p=0.04.
			Physical Function	mHAQ (0-3) mean (95% confidence interval), at baseline and 6 months (a low score indicates good performance)	There was a significant improvement in intervention mHAQ from baseline: 0.12 (0.05, 0.18) to 6 months 0.20 (0.12, 0.28) within-group p=0.06 vs control 0.22 (0.14, 0.30) to 0.24 (0.15, 0.33) within-group p=0.60 with between-group p=0.27.
Lamb et al., 2015 [25]	I: Six face-to-face sessions with a physiotherapist or an occupational therapist for seven mobility exercises and four strength or endurance exercises. C: Exercise program to usual care at home.	Three sessions to a maximum of 1.5-h contact time for 12 weeks	Disease Activity	NR	NR
			Quality of Life	SF-12 (0-100) mean (SD) at baseline, 4 and 12 months (high score indicates good performance)	There was a significant improvement (baseline: 33·8 (9·8). 4 months: 2·04 (1·01–3·08); 12 months: 1·19 (0·23–2·14)) vs control (baseline: 34·5 (9·5). 4 months: 0·91 (0·03–1·80). 12 months: 0·03 (−0·96 to 1·03)). Between-group at 4 months: p=0·0743 and 12 months: p=0·1555
			Physical Function	MHQ (0-100) mean (SD) at baseline, 4 and 12 months (high score indicates good performance)	There was a significant improvement in intervention (baseline: 52·1 (15·2). 4 months: 8·73 (6·83–10·64); 12 months: 7·93 (5·98–9·88)) vs control (baseline: 52·1 (16·4). 4 months: 4·04 (2·17-5·91). 12 months: 3·56 (1·45–5·68)). Between-group at 4 months: p=0·0001 and 12 months: p=0·0028.
Puksic et al., 2021 [26]	I: Yoga programs consist of guided relaxation (5-10 min), (5-10 min) in a supine position, breathing exercises (50-60 min), short relaxation (5 min), a special alternate nostril breathing technique (nadi shodhana pranayama) (10 min), self-inquiry meditation (5–10 min) and closing OM chant. C: weekly 60 mins lecture	Two times with 90 min per session for 12 weeks.	Disease Activity	DAS-28CRP mean (SD) score at baseline, 3 months, and 6 months (a low score indicates good performance)	Baseline 2.3 (0.8), no statistically significant change at 3 months 2.24 (0.59) and 6 months 2.28 (0.77) vs control (baseline: 2.8 (0.9), 3 months: 2.82 (0.99), 6 months: 2.46 (0.97)). Between-group p-value at 3 and 6 months: p=0.29, p=0.59.
			Quality of Life	SF-36 (0-100) mean (SD) at baseline, 3 months, and 6 months (a high score indicates good performance)	Baseline 57 (17.7), no statistically significant change at 3 months 57.92 (20.21) and 6 months 56.09 (18.95) vs control (baseline: 50.2 (20), 3 months: 49.38 (14.77), 6 months: 48.04 (18.75)). Between-group p-value at 3 and 6 months: p=0.69, p=0.14.
			Physical Function	SF-36 (0-100) mean (SD) at baseline, 3 months, and 6 months (a high score indicates good performance)	Baseline 66.5 (20.5), no statistically significant change at 3 months 70.83 (19.82) and 6 months 69.13 (19.05) vs control (baseline: 54.4 (20.4), 3 months: 59.36 (20.50), 6 months: 56.09 (24.49)). Between-group p-value at 3 and 6 months: p=0.86, p=0.90.
Siqueira et al., 2017 [27]	I: Patients in the water-based aerobic group (GW) performed only exercises in the water, whereas those in the land-based aerobic group (GL) performed on land	3 times per week for 16 consecutive weeks, totaling 48 sessions.	Disease Activity	DAS-28 ESR (<2.6 to >3.21) mean (SD) at baseline, Weeks 8 and 16 (a low score indicates good performance)	There was a significant improvement in intervention (T0 (land= 3.6 (1.2); water = 3.8 (1.2), T8 (land = 3.5 (1.3); water = 3.2 (1.1), T16 (land = 3.6 (1.2); water = 3.1 (1)) vs control (T0 = 4.3 (0.9), T8 = 4 (0.9), T16 = 4.2 (0.9)).
			Quality of Life	NR	NR
			Physical Function	mHAQ (0-3) mean (SD) at baseline, Weeks 8 and 16 (a low score indicates good performance)	There was a significant improvement in intervention (T0 (land= 0.7 (0.5); water = 0.7 (0.5), T8 (land = 0.7 (0.6); water = 0.5 (0.4), T16 (land = 0.8 (0.6); water = 0.4 (0.4)) vs control (T0 = 0.8 (0.5), T8 = 0.9 (0.8), T16 = 1.3 (1.7)).

Effect of rehabilitation intervention on disease activity

The interventions generated variable but largely favorable effects on DAS-28 measures of disease activity. Significant changes were observed for Yi Jinjing intervention [21], with median DAS-28 values declining from 2.85 to 2.15 (p=0.001), and for aquatic aerobic [27], showing significant decreases after 16 weeks, particularly for the water-based program (3.8 to 3.1). Azeez et al. [20] reported no significant DAS-28 change over three months. However, CRP levels were significantly lowered within the intervention group (p=0.002), suggesting an anti-inflammatory benefit not captured by DAS-28 [20]. Multidisciplinary intervention and cervical stabilization training demonstrated small DAS-28 decreases within the intervention, but not the comparison, group, indicating additional benefits resulting from continued pharmacologic therapy [23,24]. Yoga demonstrated stable RA activity without significant change within the following six months, highlighting that low-intensity mind-body modalities likely have modest direct effects on RA inflammatory activity [26].

Effect of rehabilitation intervention on physical function

Function measures, like the HAQ, MHQ, Duruoz Hand Index (DHI), and the SF-36 subscale for physical function, demonstrated continuous improvements throughout most intervention studies. Exercise programs developed with emphasis on strength or endurance resulted in significant HAQ or MHQ improvements compared to the control group [20,25], while galvanic electrotherapy significantly reduced DHI disability scores [22]. Cervical stabilization training generated clinically significant HAQ improvements within six weeks, while aquatic aerobic exercise was associated with further HAQ score declines than was training on land [23,27]. Even for those studies without significant disease activity changes, for example, yoga demonstrated small but positive tendencies for the physical function measures [26]. Independent of inflammatory activity changes, these studies suggest the capacity of rehabilitation, particularly when it comprises targeted or resistance training, to enhance the patient's physical potential.

Effect of rehabilitation intervention on quality of life

Questionnaires like the HAQ, EQ-5D-3L, RAQoL, SF-12, SF-36, and GFI were used to measure quality of life, and results favored the intervention groups. Azeez et al. [20] reported a significant gain on GFI (p=0.047), whereas Chang et al. [21] demonstrated significant HAQ score decreases, suggesting enhanced self-rated function. A significant RAQoL score increase was noted after cervical stabilization training for six weeks, with RAQoL scores reducing from 19.0 to 7.5 [23]. Lamb et al. [24] demonstrated persistent SF-12 gain at four and 12 months, whereas reported an EQ-5D-3L score increase with multidisciplinary intervention [25]. By comparison, no statistically significant yoga-improved SF-36 scores were reported [26]. However, numerous physical rehabilitation techniques benefit quality of life; modality, intensity, or functional domain-specific differences may exist.

Quality assessment of bias

Overall, the methodological quality of the included RCTs was generally strong but variable across the assessed domains. Sequence generation reported low risk of bias, with seven of eight trials, suggesting sufficient randomization processes were in place for most trials (see Table 4). Allocation concealment, however, was not uncommon to be rated as unclear or high risk for multiple studies, with four low risk, three unclear, and one high risk, indicating minimal reporting or potential procedural shortcomings in the maintenance of allocation concealment.

**Table 4:** Risk of Bias Table Using the Cochrane Collaboration’s Risk of Bias tool (RoB 1.0)

Primary Author's Last Name/Year	Sequence Generation	Allocation of Concealment	Blinding of Participants and Personnel	Blinding of Outcome Assessors	Incomplete Outcome Data	Selective Outcome Reporting	Other Sources of Bias
Azeez [20]/2021	Low	Unclear	High	High	Low	Low	Unclear
Chang [21]/2024	Low	Unclear	High	Low	Unclear	Low	Low
Dülgeroğlu [22]/2016	Unclear	High	High	High	Unclear	Unclear	High
Gulcemal [23]/2024	Low	Low	High	High	Unclear	Low	Unclear
Lahiri [24]/2022	Low	Low	High	Low	Low	Low	Low
Lamb [25]/2015	Low	Low	High	Low	Low	Low	Low
Puksic [26]/2021	Low	Low	High	Low	Low	Low	Low
Siqueira [27]/2017	Low	Unclear	Low	Low	Low	Low	Low

Blinding of participants and personnel was overwhelmingly assessed as a high risk of bias in most included studies (seven of eight). This is not unusual for rehabilitative and exercise-based trials, where blinding is often impractical or impossible. Nevertheless, blinding of outcome assessors was achieved more frequently (five low risk), enhancing the outcome measures' reliability, although several exceptions with unclear or high risk were noted [20-23].

Selective reporting and incomplete outcome data were overall well controlled, with most trials assessed as low risk for attrition and reporting bias, so the results reported are likely to be representative and complete. Some studies, however, were uncertain about risk in these areas [21-23]. Other biases were overall low risk, though two studies were judged unclear and one high risk to account for small numbers or limitations in the study design [20,22].

Overall, although the inability to blind participants represents a built-in limitation shared by all rehabilitation studies, the included trials followed acceptable standards for randomization, blinding of outcome assessment, and integrity of the data, lending confidence to the findings of the trials.

Discussion

This systematic review was performed to determine whether adding rehabilitation therapy to standard DMARD therapy in patients with RA enhances disease activity, physical function, and quality of life. There were consistent improvements in patient physical function and quality of life, whereas mixed results were achieved for disease activity [20-27]. The findings demonstrated that rehabilitation interventions had more favorable physical function and quality-of-life outcomes for the patients than standard DMARD therapy alone, and for reducing inflammatory disease activity.

The existing body of evidence highlights the promise of rehabilitation intervention as an adjunct to standard pharmacologic therapy for the treatment of RA. In diverse modalities (such as aerobic exercise, strength training, mind-body techniques, electrotherapy, and multidisciplinary therapy), patients exhibited enhancements of physical function and quality of life, with specific interventions also recording decreases in disease activity and inflammatory markers. These results concur with the escalating understanding that RA management ought to extend beyond pharmacotherapy to incorporate functional impairment, fatigue, and psychosocial well-being.

The results of this review align with current clinical practice guidelines, which recommend incorporating drug therapies for RA with physical activity and rehabilitation interventions [7,9]. For instance, the 2022 EULAR and 2021 ACR recommend routine physical activity as part of RA management to control inflammation, maintain joint function, and enhance quality of life [3,7-9]. The current review also supports these proposals and supplements them by demonstrating that, not only strength training and cardio exercises, but also mind-body therapies such as yoga and Yi Jinjing, electrotherapy, and multimodal treatment have the promise of generating clinically essential benefits. Hence, the current review also supports the relevance of rehabilitation as a cornerstone of current RA treatment, in agreement with guideline-directed treatment. Previous systematic reviews have also shown that structured exercise training, including strength and endurance training, improves joint mobility, quality of life, and muscle strength for RA patients [3,7-9]. The present review provides a broader perspective on RA rehabilitation by including more recent randomized trials of a more inclusive range of modalities. Despite adding heterogeneity, it gives a more inclusive sense of how numerous non-pharmacologic interventions may contribute to pharmacologic management. The present review contributes a recent and inclusive body of evidence that complements and contributes to prior published reviews.

Strengths and limitations

The diversity of outcome measures, interventions, and study designs hinders immediate comparison and synthesis of findings. Numerous studies had small patient numbers or short duration of follow-up, potentially limiting the ability to detect longer-term or subtle clinical improvements. In addition, the prevalence of female participants and the diversity of baseline disease duration may complicate the generalizability of findings across the broader RA population. The significant lack across most studies was the inability to blind participants and personnel, owing to the nature of the rehabilitative intervention, potentially generating a performance and detection bias. Though the blinding of outcome assessors often occurred, the subjective character of numerous patient-reported outcomes may remain subject to expectancy biases. In addition, publication bias was not assessed due to the limited number of studies. Also, whereas prior systematic reviews have indicated beneficial effects of exercise training for RA, they tended to be limited to specific modalities (i.e., aerobic training by itself) or small, elderly trials. This review provides a more comprehensive portrait of RA's adjunctive rehabilitation by including more diversified rehabilitation modalities (aerobic, mind-body, electrotherapy, multidisciplinary). This inclusiveness, by including heterogeneity within it as well, also places the results as complementary to previous reports and marks where consistent evidence accumulates.

Clinical research and implications

The findings of the current review align with existing clinical guideline guidance, such as the 2022 EULAR and 2021 ACR guidance, that emphasize the combination of physical activity and rehabilitation with DMARD treatment for RA. The present synthesis advances these guidelines by including current evidence to confirm the general recommendation for exercise and the value of multiple rehabilitative approaches, including mind-body therapies and multimodal care. The latter helps reinforce the clinical relevance of rehabilitation as an integral - not auxiliary - component of RA management. Given these limitations, future research should aim to employ larger, multicenter RCTs with more extended follow-up periods to evaluate the sustainability of rehabilitation benefits in RA. Identifying patient subgroups to benefit from specific rehabilitation modalities and investigating the intensity of non-pharmacological intervention could facilitate more personalized treatment approaches. Exploring novel technologies such as virtual reality or wearable sensors for tele-rehabilitation also promises to expand access and adherence to physical therapy programs. Additionally, the impact on healthcare utilization and assessing cost-effectiveness would inform implementation strategies in clinical practice. Clinicians should therefore consider structured rehabilitation not only as supportive care, but as an essential therapeutic intervention that enhances quality of life, functional autonomy, and perhaps reduces disease burden as an adjuvant to drug treatment.

## Conclusions

This systematic review supports incorporating rehabilitation therapy into routine RA treatment programs. However, few high-quality studies, heterogeneity of interventions, and methodology limitations, including risk of bias and short follow-up periods, render these findings uncertain. Individual modalities, intensities, and delivery models of rehabilitation were not conclusively recommended. Still, the overall patient-centered benefit pattern supports consideration of patient-specific exercise or function training programs at the clinical level. Clinicians should weigh patient-specific needs, limitations, and wishes against inclusion of rehabilitation approaches.

Future multicenter, randomized, high-quality investigations will need to ascertain optimal intervention durations, intensities, and types, assess possible disease activity impacts, and demonstrate sustainability and cost-effectiveness on a long-term basis. Such investigations will yield practical evidence-based, pragmatic guideline recommendations on incorporating rehabilitative measures into optimal RA management.

## Appendices

**Table 5:** Database Search Syntax and Strategies for Rheumatoid Arthritis Treatment Outcomes

Database	Date Searched	Search Syntax	Result Retrieved
PubMed	July 21, 2025	("arthritis, rheumatoid"[MeSH Terms] OR "rheumatoid arthritis"[tiab]) AND ("rehabilitation"[MeSH Terms] OR "exercise therapy"[MeSH Terms] OR "physical therapy modalities"[MeSH Terms] OR rehabilitation[tiab] OR "physical therapy"[tiab] OR "exercise"[tiab]) AND ("antirheumatic agents"[MeSH Terms] OR DMARD*[tiab] OR "biologic DMARD*"[tiab] OR "csDMARD*"[tiab] OR methotrexate[tiab] OR leflunomide[tiab] OR abatacept[tiab] OR adalimumab[tiab] OR etanercept[tiab]) AND ("quality of life"[MeSH Terms] OR "functional status"[tiab] OR HAQ[tiab] OR "disease activity"[tiab] OR DAS28[tiab]) AND ("randomized controlled trial"[Publication Type] OR "systematic review"[pt] OR "meta-analysis"[pt]) AND english[lang] AND ("2013/01/01"[PDAT] : "2025/07/20"[PDAT])	19
Embase	July 21, 2025	('arthritis, rheumatoid'/exp OR 'arthritis, rheumatoid':ti,ab OR 'rheumatoid arthritis':ti,ab) AND ('rehabilitation'/exp OR rehabilitation:ti,ab OR 'physical therapy'/exp OR 'physical therapy':ti,ab OR 'exercise therapy'/exp OR exercise:ti,ab) AND ('antirheumatic agent'/exp OR dmard*:ti,ab OR 'biologic dmard*':ti,ab OR 'csdmard*':ti,ab OR methotrexate:ti,ab OR leflunomide:ti,ab OR abatacept:ti,ab OR adalimumab:ti,ab OR etanercept:ti,ab) AND ('quality of life'/exp OR 'quality of life':ti,ab OR 'functional status':ti,ab OR haq:ti,ab OR 'disease activity':ti,ab OR das28:ti,ab) AND ([randomized controlled trial]/lim OR [systematic review]/lim OR [meta analysis]/lim) AND [english]/lim Filters: year of publication: 2013-2025	171
Cochrane Library	July 21, 2025	("arthritis, rheumatoid" OR "rheumatoid arthritis") AND (rehabilitation OR "physical therapy" OR exercise) AND ("antirheumatic agents" OR DMARD OR "biologic DMARD" OR "csDMARD" OR methotrexate OR leflunomide OR abatacept OR adalimumab OR etanercept) AND ("quality of life" OR "functional status" OR HAQ OR "disease activity" OR DAS28)	44
